# Associations between sarcopenia (defined by low muscle mass), inflammatory markers, and all-cause mortality in older adults: mediation analyses in a large U.S. NHANES community sample, 1999–2006

**DOI:** 10.3389/fmed.2025.1515839

**Published:** 2025-07-10

**Authors:** Jia Liu, Fuchun Zhang

**Affiliations:** Department of Geriatrics, Peking University Third Hospital, Beijing, China

**Keywords:** sarcopenia, National Health and Nutrition Examination Survey (NHANES), inflammation, older adults, mortality

## Abstract

**Background:**

Sarcopenia is linked to increased mortality, but the specific role of inflammation in sarcopenia-related mortality remains poorly understood. This study aims to integrate various inflammatory biomarkers to develop an inflammation prognostic score (IPS) within the large, representative NHANES cohort. It also explores the association between sarcopenia, inflammatory markers, and mortality, and investigates whether inflammation mediates this relationship.

**Methods:**

This study analyzed data from NHANES (1999–2006) on 3,544 participants aged 65 and older, with mortality follow-up through December 31, 2019, using death records from the National Death Index (NDI). Statistical analyses accounted for complex survey design and multiple imputation for missing data. Sarcopenia was defined using appendicular skeletal mass (ASM) adjusted for body mass index (BMI). Cox regression assessed the association between sarcopenia, inflammatory markers, and all-cause mortality. The IPS was developed using LASSO regression, and mediation analysis was conducted to assess whether inflammatory markers mediate the relationship between sarcopenia and mortality.

**Results:**

Among 3,544 elderly participants, sarcopenia was present in 25.4%, with a 66.6% overall mortality rate during the follow-up period. Multivariate Cox regression confirmed that sarcopenia is an independent risk factor for mortality [Hazard ratio (HR) = 1.235–1.281, *P* < 0.001]. Inflammatory markers were significantly associated with all-cause mortality. The IPS showed a clear trend of increasing mortality risk across quartiles, with HR reaching 2.044 in Q4 (*P* < 0.001). Mediation analysis showed that IPS mediated 20.8% of the relationship between sarcopenia and mortality, with the mediating effect remaining significant after adjusting for confounders.

**Conclusion:**

This study confirms the association between sarcopenia and increased mortality risk, with inflammation as a key mediating factor, highlighting its role in sarcopenia-related mortality.

## 1 Introduction

As global aging accelerates, health concerns among older adults have become a major public health challenge. Population aging has emerged as one of the most pressing global public health challenges, with profound implications for health systems, long-term care, and chronic disease management (1, 2). Sarcopenia, an age-related condition defined by the loss of muscle mass, strength and function, is associated with adverse clinical outcomes such as falls, fractures, frailty, hospitalization, and an increased risk of mortality. Numerous epidemiological studies have confirmed that individuals with sarcopenia exhibit significantly higher risks of both short- and long-term mortality, even after adjusting for age, comorbidities, and functional status. This association highlights the clinical importance of identifying and addressing sarcopenia as a means to reduce premature death in aging populations (3, 4). Sarcopenia can be influenced by malnutrition, lack of physical activity, and disease-related reduced mobility, with inflammation also being a key factor in its development (3). Diseases that trigger inflammation, such as cancer and organ failure, along with age-related chronic low-grade inflammation, are key factors contributing to the onset of sarcopenia. Key inflammatory molecules, including TNF-α, IL-6, and IL-1β, activate pathways like NF-κB and inhibit the PI3K/AKT/mTOR axis, leading to reduced protein synthesis and increased muscle degradation (5, 6). Chronic inflammation also disrupts mitochondrial function, impairs autophagy, and weakens the regenerative capacity of muscle satellite cells. In addition, immune aging, fat infiltration into muscle, and gut microbiota imbalance can further exacerbate systemic inflammation and accelerate sarcopenia progression (7, 8).

From a pathophysiological perspective, sarcopenia is closely associated with chronic inflammation, which is linked to poor prognosis. Elevated inflammation predicts all-cause mortality in older adults, independent of other risk factors (9). Factors such as social conditions, environmental influences, and lifestyle choices also contribute to systemic chronic inflammation, which is further associated with cardiovascular diseases, cancer, diabetes, neurodegenerative disorders, and other major causes of disability and mortality (10). Traditional biomarkers for assessing inflammation include white blood cell count (WBC), neutrophil count (NEU), lymphocyte count (LYM), and monocyte count (MNO). These markers respond to acute inflammation from bacterial or viral infections, reflecting disease severity and prognosis. Increasing research highlights red cell distribution width (RDW) as a marker of systemic inflammation and an independent predictor of poor prognosis and mortality in conditions such as cardiovascular disease, cancer, and sepsis (11–13). Alkaline phosphatase (ALP), originating primarily from the liver, bones, and intestines, is also involved in cardiovascular disease by promoting vascular calcification and endothelial dysfunction, and it is significantly associated with increased mortality (14, 15). Lactate dehydrogenase (LDH), a cytoplasmic enzyme, plays a key role in metabolism. Elevated serum LDH, indicative of cell damage or death, is linked to inflammation and poor clinical outcomes (16, 17). C-reactive protein (CRP), an acute-phase reactant expressed by hepatocytes, serves as an inflammatory marker and is associated with poor prognosis (18). Beyond these singular markers, composite inflammatory indices provide a more comprehensive view of systemic inflammation. The neutrophil-to-lymphocyte ratio (NLR) is defined as NEU divided by LYM. The derived neutrophil-to-lymphocyte ratio (d-NLR) is calculated as NEU divided by (WBC minus NEU). The monocyte-to-lymphocyte ratio (MLR) is the ratio of MNO to LYM. The systemic inflammation response index (SIRI) is calculated as NEU multiplied by MNO, then divided by LYM. NLR, d-NLR, MLR, and SIRI have been shown to be associated with poor prognosis in various conditions, reflecting the overall state of systemic inflammation (19–22).

Although sarcopenia is known to be associated with inflammation and increased mortality, the specific role of inflammation in sarcopenia-related mortality remains poorly understood. Current research provides limited insights into the combined impact of multiple inflammatory markers in this context. This study aims to integrate various inflammatory biomarkers to develop an inflammation prognostic score (IPS) within the large, representative NHANES cohort, and to investigate the role of inflammation in sarcopenia-related mortality.

Therefore, this study aims to investigate: (1) the association between sarcopenia, inflammatory markers, and mortality; and (2) whether inflammatory markers mediate the relationship between sarcopenia and mortality. We hypothesize that (1) sarcopenia, inflammatory markers, and mortality are interconnected; and (2) inflammatory markers partially mediate the effect of sarcopenia on all-cause mortality.

## 2 Materials and methods

### 2.1 Study design and population

The National Health and Nutrition Examination Survey (NHANES), conducted by the Centers for Disease Control and Prevention (CDC), is a nationwide survey designed to obtain a representative sample of the U.S. population. Using a complex, stratified sampling method, NHANES aims to assess the health and nutritional status of U.S. adults and children. The survey includes both interview and physical examination components, collecting comprehensive demographic, socioeconomic, dietary, and health-related data. Further details are available at the CDC website (https://www.cdc.gov/nchs/nhanes/). The study protocols were approved by the National Center for Health Statistics (NCHS) Research Ethics Review Board (ERB) and are reviewed annually. All participants provided written informed consent.

**Figure 1:**
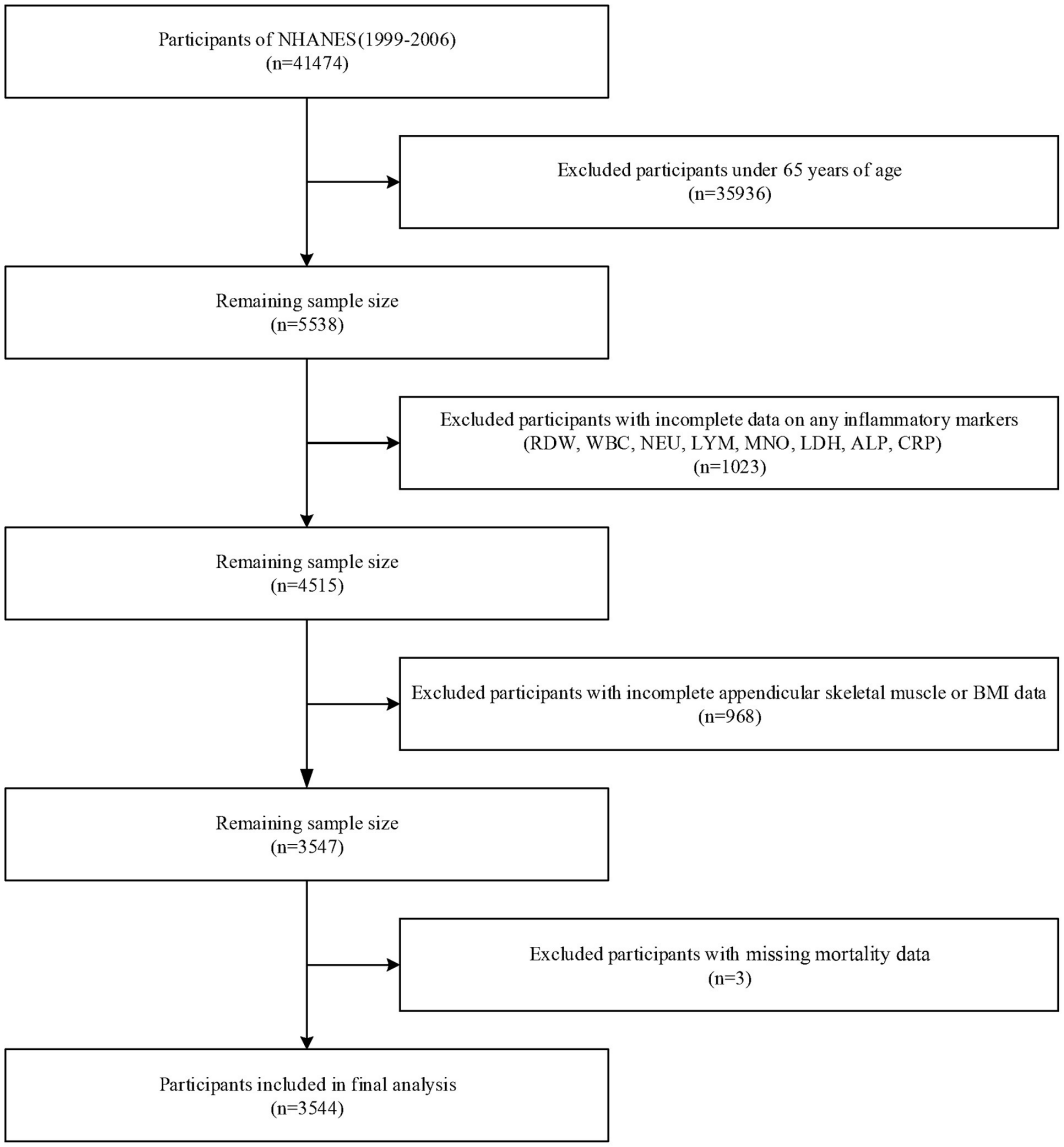
Flowchart of participant selection.

The study originally included 41,474 participants from four NHANES cycles (1999–2006). Participants under 65 years of age (*n* = 35,936) were excluded, along with those with incomplete data on any of the eight inflammatory markers (RDW, WBC, NEU, LYM, MNO, LDH, ALP, and CRP; *n* = 1,023), incomplete appendicular skeletal muscle and body mass index (BMI) data (*n* = 968), and missing mortality data (*n* = 3). Ultimately, 3,544 participants were included in the final analysis (Figure 1).

### 2.2 Sarcopenia definition

BMI was assessed at the mobile examination center by dividing weight in kilograms by the square of height in meters (kg/m^2^). Whole body Dual-energy X-ray absorptiometry (DXA) scans were conducted at the NHANES mobile examination center. Participants were excluded from DXA if they met any of the following criteria: self-reported use of radiographic contrast agents within the past 7 days, nuclear medicine procedures within the past 3 days, or a weight exceeding 136 kg or height over 196 cm, due to DXA table limitations. Whole body DXA scans were performed using a Hologic QDR-4500A densitometer. Appendicular Skeletal Mass (ASM), an accepted proxy for skeletal muscle mass, was calculated by summing the lean mass (excluding bone mineral content) of both legs and arms as measured by DXA. According to the sarcopenia criteria proposed by the Foundation for the National Institutes of Health (FNIH), the skeletal muscle index (SMI) is calculated as appendicular skeletal muscle mass divided by body mass index (ASM/BMI), that is, ASM (kg)/BMI (kg/m^2^). The cut-off values for sarcopenia are < 0.789 for men and < 0.512 for women (23). Functional indicators such as grip strength or gait speed were not available in the NHANES 1999–2006 dataset, and therefore could not be incorporated into the definition.

### 2.3 Definition of inflammatory markers

The inflammatory markers analyzed in this study include WBC, NEU, LYM, MNO, RDW, LDH, ALP, and CRP. Blood samples were collected after fasting, with complete blood counts performed at the mobile examination center, while other parameters were measured from refrigerated or frozen samples sent to a central laboratory. Complete blood counts, including WBC, NEU, LYM, MNO, and RDW, were measured using the Beckman Coulter MAXM analyzer. LDH and ALP were analyzed with the Hitachi Model 704 multichannel analyzer, and CRP was quantified using the Behring Nephelometer.

Additional inflammatory indices were calculated as follows (24): NLR = NEU (10^9^/L)/LYM (10^9^/L); dNLR = NEU (10^9^/L)/[WBC (10^9^/L) – NEU (10^9^/L)]; LMR = LYM (10^9^/L)/MNO (10^9^/L); and SIRI = NEU (10^9^/L) ^*^ MNO (10^9^/L)/LYM (10^9^/L).

### 2.4 Outcome definition

The NCHS has connected survey data with death certificate records from the National Death Index (NDI) to offer mortality follow-up information from the date of survey participation until December 31, 2019. The main outcome of this study is all-cause mortality. We used the full follow-up period to capture long-term mortality outcomes, consistent with standard NHANES survival analyses. This approach ensures sufficient event numbers and aligns with the time-to-event structure of Cox models.

### 2.5 Covariates

Covariates were selected based on previous literature. Demographic variables include gender, age, race (Hispanic; Non-Hispanic White; Non-Hispanic Black; Non-Hispanic Asian; Other Race), education level (below high school, high school, and above high school), and poverty income ratio (PIR), categorized as poor (PIR < 1) or not poor (PIR ≥ 1). Physical activity level was classified as inactive (< 150 min of moderate or 75 min of vigorous activity per week), moderate (150–300 min of moderate or 75–150 min of vigorous activity per week), and active (more than 300 min of moderate or 150 min of vigorous activity per week) (25). Smoking status was categorized as never smoked (< 100 cigarettes in a lifetime), ever smoked (more than 100 cigarettes but not currently smoking), and currently smoking. Alcohol consumption was classified as no drink, moderate drink (< 2 drinks per day for men, < 1 drink per day for women), and heavy drink (two or more drinks per day for men, one or more drinks per day for women). Hypertension was defined as mean systolic blood pressure ≥140 mmHg, mean diastolic blood pressure ≥90 mmHg, self-reported hypertension, or current antihypertensive medication use. Diabetes was defined as self-reported diabetes, glycated hemoglobin ≥6.5%, or fasting blood glucose ≥7 mmol/L. Chronic conditions such as congestive heart failure, coronary heart disease, stroke, and cancer were also included. Laboratory data included urinary albumin (Ualb) and blood samples for total cholesterol, triglycerides, creatinine, aspartate aminotransferase (AST), alanine aminotransferase (ALT), albumin (ALB), red blood cell count (RBC), and hemoglobin levels (HGB). Details on laboratory procedures, data processing, quality control, and analysis are available on the NHANES website (http://wwwn.cdc.gov/nchs/nhanes/).

### 2.6 Statistical analysis

Statistical analyses were conducted using R software (version 4.2.1, https://www.R-project.org/). In accordance with NHANES analysis guidelines, we accounted for the complex sampling design and applied sampling weights. Missing data were handled via multiple imputation using the random forest algorithm in the “mice” package, generating five imputed datasets for separate analysis. The results were then combined using Rubin's rules with the “mitools” package. Descriptive statistics are reported as median [interquartile range (IQR)] for continuous variables and as counts with weighted proportions for categorical variables. Baseline characteristics were compared between participants with and without sarcopenia. Group comparisons were performed using design-adjusted chi-square tests for categorical variables and design-based *t*-tests for continuous variables.

Cox regression was used to assess the association between sarcopenia and all-cause mortality. Survival time was defined from the date of NHANES interview to the date of death or censoring (December 31, 2019). Hazard ratios (HRs) and 95% confidence intervals (CIs) were estimated. A series of models with progressive adjustment for demographic, clinical, lifestyle, and laboratory variables were applied, as detailed in the covariates section. The prognostic performance of 12 inflammatory markers, treated as continuous variables, was evaluated using time-dependent ROC analysis, with AUCs calculated at 3, 5, and 7 years. Optimal cut-off values for the 12 inflammatory markers predicting mortality were identified using the R package “survminer,” after which continuous inflammatory markers were converted into binary variables (1 for above and 0 for below the threshold), and their associations with mortality were further evaluated using Cox regression. To capture the combined inflammatory burden, we developed the IPS using LASSO-Cox regression implemented via the R package “glmnet.” LASSO was applied to automatically select the most prognostically informative markers while minimizing multicollinearity, with the optimal penalty parameter determined through 10-fold cross-validation (26, 27). The final IPS was calculated for each participant as a weighted sum of the selected markers, with weights derived from the corresponding LASSO-Cox coefficients. This data-driven approach ensured that only markers with independent contributions to mortality risk were retained. Cox regression models were used to estimate all-cause mortality risk across the IPS quartiles (25th, 50th, and 75th percentiles), with trend tests conducted to assess significance. Multivariable linear regression analyzed the correlation between sarcopenia and inflammatory markers.

Mediation analysis was performed to assess whether inflammatory markers mediate the relationship between sarcopenia and mortality, using 1,000 bootstrap samples. The average causal mediation effect (ACME), the proportion of the mediation effect, and the *P*-value for the mediation were reported. A *P*-value of < 0.05 (two-tailed) was considered statistically significant.

## 3 Results

### 3.1 Baseline characteristics

A total of 3,544 older adults were included in the analysis, of whom 1,062 (25.4%) met the criteria for sarcopenia. During the follow-up period, the overall mortality rate was 66.6%. Table 1 presents the baseline characteristics of participants stratified by sarcopenia status. Compared to those without sarcopenia, participants with sarcopenia were significantly older and had higher levels of inflammatory markers, including RDW, WBC, NEU, CRP, NLR, dNLR, and SIRI (all *P* < 0.05). In contrast, the LMR level was significantly lower in the sarcopenia group (*P* < 0.001). Sarcopenic individuals also had significantly higher BMI and a greater proportion of males, individuals with lower educational attainment, and lower physical activity levels. Regarding comorbidities, diabetes mellitus (30 vs. 18%, *P* < 0.001), heart failure (11 vs. 7%, *P* = 0.001), and coronary artery disease (16 vs. 11%, *P* = 0.005) were more prevalent in the sarcopenia group. During the follow-up period, the weighted all-cause mortality rate was significantly higher among participants with sarcopenia than those without (75 vs. 64%, *P* < 0.001).

**Table 1:** Baseline characteristics of participants with and without sarcopenia.

**Variable**	**Sarcopenia group (*n* = 1062, 25.4%)**	**Non-sarcopenia group (*n* = 2482, 74.6%)**	***p*-value**
Age (years)	73 (69, 80)	71 (67, 77)	<0.001^*^
Male, *n* (%)	605 (52)	1174 (40)	<0.001^*^
**Race**			
Mexican American, *n* (%)	339 (6)	321 (2)	0.004^*^
Non-Hispanic White, *n* (%)	600 (81)	1567 (84)	
Non-Hispanic Black, *n* (%)	51 (3)	469 (9)	
Other, *n* (%)	72 (10)	125 (5)	
PIR, poor, *n* (%)	201 (12)	343 (10)	0.107
**Education**			
Below high school, *n* (%)	541 (36)	944 (28)	<0.001^*^
High school, *n* (%)	231 (30)	610 (29)	
Above high school, *n* (%)	290 (34)	928 (43)	
**Physical activity level**			
Inactive, *n* (%)	677 (61)	1264 (48)	<0.001^*^
Moderate, *n* (%)	130 (14)	370 (16)	
Active, *n* (%)	255 (25)	848 (37)	
**Alcohol consumption**			
No drink, *n* (%)	300 (29)	624 (26)	0.255
Moderate drink, *n* (%)	710 (66)	1721 (68)	
Heavy drink, *n* (%)	52 (5)	137 (6)	
**Smoking status**			
Never smoke, *n* (%)	502 (48)	1197 (49)	0.165
Ever smoke, *n* (%)	468 (44)	1021 (41)	
Now smoke, *n* (%)	92 (8)	264 (10)	
BMI (kg/m^2^)	29.48 (26.49, 33.29)	26.59 (23.90, 30.03)	<0.001^*^
**Inflammatory markers**			
RDW (%)	12.9 (12.4, 13.5)	12.7 (12.3, 13.3)	<0.001^*^
WBC (× 10^9^/L)	7.1 (6.0, 8.4)	6.7 (5.6, 7.9)	0.01^*^
NEU (× 10^9^/L)	4.4 (3.5, 5.3)	4.0 (3.2, 4.9)	<0.001^*^
LYM (× 10^9^/L)	1.8 (1.4, 2.2)	1.8 (1.4, 2.3)	0.523
MNO (× 10^9^/L)	0.6 (0.5, 0.7)	0.6 (0.5, 0.7)	0.004^*^
LDH (U/L)	142.0 (124.0, 164.0)	141.0 (124.0, 163.0)	0.510
ALP (U/L)	72.0 (60.0, 89.0)	71.0 (58.0, 88.0)	0.626
CRP (mg/dl)	0.33 (0.16, 0.68)	0.24 (0.11, 0.49)	0.002^*^
NLR	2.42 (1.76, 3.20)	2.18 (1.62, 2.93)	<0.001^*^
dNLR	1.63 (1.27, 2.04)	1.51 (1.16, 1.92)	<0.001^*^
LMR	3.00 (2.38, 4.00)	3.33 (2.57, 4.20)	<0.001^*^
SIRI	1.38 (0.94, 2.06)	1.20 (0.82, 1.71)	<0.001^*^
**Other Laboratory Tests**			
Ualb (mg/L)	11.0 (5.3, 31.2)	8.4 (4.0, 19.5)	0.098
Total cholesterol (mmol/L)	5.17 (4.53, 5.95)	5.35 (4.68, 6.05)	0.05
Triglycerides (mmol/L)	1.49 (1.11, 2.08)	1.43 (1.02, 2.04)	0.083
Creatinine (μmol/L)	79.56 (70.70, 97.24)	79.56(70.70, 97.24)	0.07
AST (U/L)	23.0 (20.0, 27.0)	23.0 (20.0, 27.0)	0.064
ALT (U/L)	20.0 (16.0, 25.0)	19.0 (16.0, 24.0)	0.113
ALB (U/L)	42.0 (40.0, 44.0)	42.0 (41.0, 44.0)	<0.001^*^
RBC (× 10^12^/L)	4.61 (4.22, 4.93)	4.58 (4.28, 4.90)	0.969
HGB (g/L)	14.3 (13.3, 15.1)	14.2 (13.3, 15.1)	0.768
HBP, *n* (%)	788 (74)	1791 (72)	0.297
DM, *n* (%)	334 (30)	529 (18)	<0.001^*^
**Comorbidities**			
HF, *n* (%)	110 (11)	174 (7)	0.001^*^
CAD, *n* (%)	131 (16)	259 (11)	0.005^*^
Cancer, *n* (%)	183 (21)	509 (23)	0.225
Stroke, *n* (%)	108 (10)	180 (7)	0.06
All-cause mortality, *n* (%)	790 (75)	1691 (64)	<0.001^*^

^*^Indicate statistical significance.

Data are presented as medians with interquartile ranges (IQRs) for continuous variables, and as unweighted counts with weighted percentages for categorical variables.

RDW, red cell distribution width; WBC, white blood cell count; NEU, neutrophil count; LYM, lymphocyte count; MNO, monocyte count; LDH, lactate dehydrogenase; ALP, alkaline phosphatase; CRP, C-reactive protein; NLR, neutrophil-to-lymphocyte ratio; d-NLR, derived neutrophil-to-lymphocyte ratio; LMR, lymphocyte-to-monocyte ratio; SIRI, systemic inflammation response index; BMI, body mass index; Ualb, urinary albumin; AST, aspartate aminotransferase; ALT, alanine aminotransferase; ALB, albumin; RBC, red blood cell count; HGB, hemoglobin; PIR, poverty income ratio; HBP, hypertension; DM, diabetes; HF, congestive heart failure; CAD, coronary heart disease.

### 3.2 Association between sarcopenia and mortality

Multivariate Cox regression analysis showed a significant association between sarcopenia and mortality. In unadjusted Model 1, the hazard ratio (HR) for mortality in sarcopenia patients was 1.450 [95% confidence interval (CI): 1.295–1.617, *P* < 0.001]. This association remained significant after adjusting for confounders in Models 2–4, with adjusted HRs ranging from 1.235 to 1.281 (*P* < 0.001 for all models). These findings indicate that sarcopenia is an independent risk factor for mortality, even after controlling for various demographic, clinical, and biochemical factors. Detailed results are presented in Table 2.

**Table 2:** Multivariate Cox regression analysis of the association between sarcopenia and all-cause mortality.

**Model**	**HR**	**95% CI**	***p*-value**
Model 1	1.450	1.295, 1.617	<0.001^*^
Model 2	1.281	1.157, 1.418	<0.001^*^
Model 3	1.247	1.120, 1.388	<0.001^*^
Model 4	1.235	1.107, 1.377	<0.001^*^

^*^Indicate statistical significance.

HR, hazard ratio; CI, confidence interval.

Model 1: adjusted for none; Model 2: adjusted for age, race, PIR, and education; Model 3: adjusted for age, race, PIR, education, BMI, physical activity level, hypertension, diabetes, heart failure, coronary heart disease, cancer, stroke, stroking status, and alcohol consumption; Model 4: adjusted for age, race, PIR, education, BMI, physical activity level, hypertension, diabetes, heart failure, coronary heart disease, cancer, stroke, stroking status, alcohol consumption, urinary albumin, total cholesterol, triglycerides, creatinine, AST, ALT, ALB, RBC, HGB.

### 3.3 Inflammatory markers and all-cause mortality

The prognostic performance of 12 inflammatory markers was assessed by time-dependent ROC analysis, with AUCs calculated at 3, 5, and 7 years (Table 3). Optimal cut-off values for each marker were determined using the maximally selected rank statistic were also listed in the table. Among the markers, RDW, NLR, and SIRI demonstrated the highest discriminative ability for all-cause mortality, with 5-year AUCs of 0.647 (95% CI: 0.623–0.672), 0.635 (95% CI: 0.609–0.660), and 0.639 (95% CI: 0.614–0.663), respectively. Overall, the AUC values of most markers ranged between 0.55 and 0.65, suggesting modest but potentially clinically useful prognostic power. Notably, LMR and LYM yielded AUCs consistently below 0.5, reflecting an inverse association with mortality—higher levels of these markers were associated with lower risk of death. These findings indicate that certain inflammatory markers, particularly RDW, NLR, and SIRI, may provide useful prognostic information regarding long-term mortality risk in older adults.

**Table 3:** Optimal cut-off values and time-dependent AUCs (95% CIs) of inflammatory markers for predicting all-cause mortality.

**Variable**	**Cut-off value**	**3-year AUC (95% CI)**	**5-year AUC (95% CI)**	**7-year AUC (95% CI)**
RDW (%)	13.20	0.643 (0.611, 0.676)	0.647 (0.623, 0.672)	0.638 (0.617, 0.659)
WBC (× 10^9^/L)	8.10	0.555 (0.520, 0.590)	0.549 (0.523, 0.575)	0.565 (0.543, 0.587)
NEU (× 10^9^/L)	3.30	0.586 (0.551, 0.620)	0.588 (0.562, 0.613)	0.594 (0.573, 0.616)
LYM (× 10^9^/L)	1.30	0.410 (0.377, 0.444)	0.400 (0.374, 0.425)	0.431 (0.408, 0.453)
MNO (× 10^9^/L)	0.60	0.559 (0.525, 0.593)	0.547 (0.522, 0.572)	0.561 (0.539, 0.582)
LDH (U/L)	166	0.568 (0.535, 0.602)	0.567 (0.542, 0.592)	0.565 (0.543, 0.587)
ALP (U/L)	103	0.567 (0.534, 0.600)	0.551 (0.526, 0.577)	0.548 (0.526, 0.570)
CRP (mg/dl)	0.80	0.596 (0.562, 0.629)	0.565 (0.539, 0.591)	0.554 (0.532, 0.576)
NLR	2.93	0.625 (0.590, 0.659)	0.635 (0.609, 0.660)	0.618 (0.596, 0.640)
dNLR	1.54	0.607 (0.572, 0.642)	0.616 (0.591, 0.642)	0.603 (0.581, 0.625)
LMR	1.54	0.370 (0.338, 0.403)	0.371 (0.346, 0.395)	0.386 (0.365, 0.408)
SIRI	1.34	0.636 (0.602, 0.669)	0.639 (0.614, 0.663)	0.631 (0.610, 0.652)

RDW, red cell distribution width; WBC, white blood cell count; NEU, neutrophil count; LYM, lymphocyte count; MNO, monocyte count; LDH, lactate dehydrogenase; ALP, alkaline phosphatase; CRP, C-reactive protein; NLR, neutrophil-to-lymphocyte ratio; d-NLR, derived neutrophil-to-lymphocyte ratio; LMR, lymphocyte-to-monocyte ratio; SIRI, systemic inflammation response index; AUC, area under the curve; CI, confidence interval.

Cox regression models were used to assess the relationship between inflammatory markers and all-cause mortality across four progressively adjusted models: Model 1 included each inflammatory marker alone; Model 2 adjusted for demographic factors (age, race, PIR, and, education); Model 3 further adjusted for clinical factors (BMI, physical activity, hypertension, diabetes, heart failure, coronary heart disease, cancer, stroke, smoking, and alcohol consumption); and Model 4 additionally adjusted for biochemical factors (Ualb, total cholesterol, triglycerides, creatinine, AST, ALT, ALB, RBC, and HGB). In multivariate Cox regression, all 12 inflammatory markers were significantly associated with all-cause mortality (*P* < 0.001; Table 4).

**Table 4:** Multivariate Cox regression analysis of inflammatory markers and all-cause mortality.

**Variable**	**Model 1**		**Model 2**		**Model 3**		**Model 4**	
	**HR (95% CI)**	***p*** **-value**	**HR (95% CI)**	***p*** **-value**	**HR (95% CI)**	***p*** **-value**	**HR (95% CI)**	***p*** **-value**
RDW (%)	1.869 (1.718, 2.033)	<0.001^*^	1.706 (1.576, 1.846)	<0.001^*^	1.565 (1.431, 1.711)	<0.001^*^	1.552 (1.401, 1.720)	<0.001^*^
WBC (× 10^9^/L)	1.392(1.241, 1.560)	<0.001^*^	1.384 (1.248, 1.535)	<0.001^*^	1.229 (1.099, 1.375)	<0.001^*^	1.233 (1.101, 1.381)	<0.001^*^
NEU (× 10^9^/L)	1.477 (1.320, 1.654)	<0.001^*^	1.352 (1.225, 1.492)	<0.001^*^	1.219 (1.112, 1.336)	<0.001^*^	1.236 (1.124, 1.358)	<0.001^*^
LYM (× 10^9^/L)	0.691 (0.616, 0.776)	<0.001^*^	0.712 (0.653, 0.777)	<0.001^*^	0.713 (0.659, 0.773)	<0.001^*^	0.740 (0.680, 0.805)	<0.001^*^
MNO (× 10^9^/L)	1.356 (1.228, 1.496)	<0.001^*^	1.247 (1.137, 1.367)	<0.001^*^	1.167 (1.061, 1.284)	0.001^*^	1.157 (1.044, 1.283)	0.006^*^
LDH (U/L)	1.504 (1.376, 1.644)	<0.001^*^	1.324 (1.217, 1.441)	<0.001^*^	1.331 (1.203, 1.472)	<0.001^*^	1.332 (1.198, 1.481)	<0.001^*^
ALP (U/L)	1.431 (1.248, 1.641)	<0.001^*^	1.345 (1.195, 1.515)	<0.001^*^	1.223 (1.060, 1.413)	0.006^*^	1.183 (1.028, 1.363)	0.019^*^
CRP (mg/dl)	1.413 (1.211, 1.649)	<0.001^*^	1.506 (1.302, 1.743)	<0.001^*^	1.407 (1.217, 1.626)	<0.001^*^	1.334 (1.140, 1.561)	<0.001^*^
NLR	1.719 (1.546, 1.912)	<0.001^*^	1.594 (1.442, 1.762)	<0.001^*^	1.504 (1.350, 1.675)	<0.001^*^	1.495 (1.341, 1.667)	<0.001^*^
dNLR	1.468 (1.351, 1.594)	<0.001^*^	1.387 (1.263, 1.523)	<0.001^*^	1.290 (1.169, 1.424)	<0.001^*^	1.282 (1.169, 1.407)	<0.001^*^
LMR	0.619 (0.560, 0.684)	<0.001^*^	0.690 (0.614, 0.775)	<0.001^*^	0.717 (0.643, 0.800)	<0.001^*^	0.742 (0.666, 0.828)	<0.001^*^
SIRI	1.615 (1.444, 1.805)	<0.001^*^	1.482 (1.328, 1.653)	<0.001^*^	1.365 (1.227, 1.518)	<0.001^*^	1.334 (1.195, 1.489)	<0.001^*^

^*^Indicate statistical significance.

RDW, red cell distribution width; WBC, white blood cell count; NEU, neutrophil count; LYM, lymphocyte count; MNO, monocyte count; LDH, lactate dehydrogenase; ALP, alkaline phosphatase; CRP, C-reactive protein; NLR, neutrophil-to-lymphocyte ratio; d-NLR, derived neutrophil-to-lymphocyte ratio; LMR, lymphocyte-to-monocyte ratio; SIRI, systemic inflammation response index; HR, hazard ratio; CI, confidence interval.

Model 1: adjusted for none; Model 2: adjusted for age, race, PIR, and education; Model 3: adjusted for age, race, PIR, education, BMI, physical activity level, hypertension, diabetes, heart failure, coronary heart disease, cancer, stroke, stroking status, and alcohol consumption; Model 4: adjusted for age, race, PIR, education, BMI, physical activity level, hypertension, diabetes, heart failure, coronary heart disease, cancer, stroke, stroking status, alcohol consumption, urinary albumin, total cholesterol, triglycerides, creatinine, AST, ALT, ALB, RBC, HGB.

**Figure 2:**
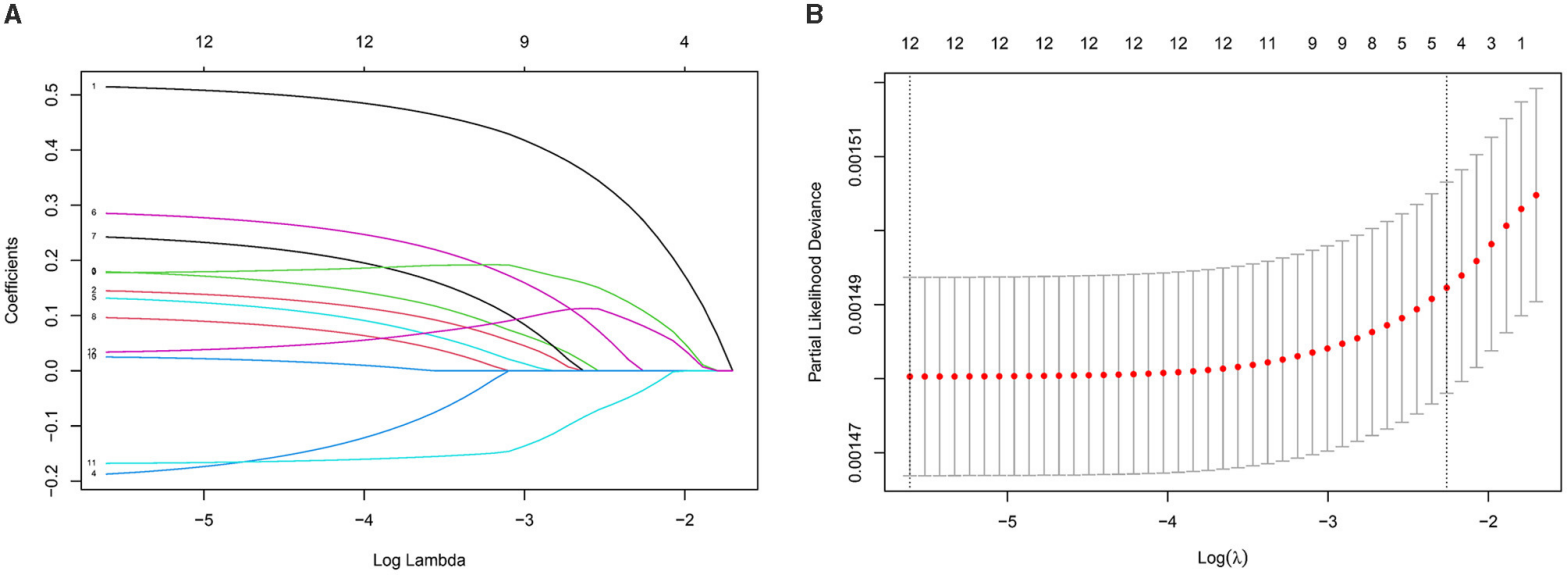
Development of the inflammatory prognostic score (IPS) using LASSO regression with 10-fold cross-validation. **(A)** Coefficient profiles of the 12 inflammatory markers. Each curve represents the change in a marker's regression coefficient as the regularization parameter lambda (λ) increases. As λ increases, coefficients progressively shrink toward zero, indicating the variable selection process of the LASSO model. **(B)** Ten-fold cross-validation for selecting the optimal λ. The *x*-axis shows log(λ), and the *y*-axis represents the partial likelihood deviance. The left dashed line indicates the λ with the minimum deviance; the right dashed line indicates a simpler model within one standard error.

Using LASSO Cox regression, we developed an IPS scoring system for elderly patients, calculated as: IPS = RDW × 0.514 + WBC × 0.145 + NEU × 0.180 + LYM × (−0.187) + MNO × 0.132 + LDH × 0.285 + ALP × 0.242 + CRP × 0.096 + NLR × 0.178 + dNLR × 0.025 + LMR × (−0.168) + SIRI × 0.034. To assess the relationship between IPS and mortality risk, the study population was divided into quartiles based on IPS values: Q1 (≤ 0.070), Q2 (0.070–0.184), Q3 (0.184–0.549), and Q4 (>0.549; Figure 2). To further evaluate the prognostic performance of the IPS as a continuous variable, we conducted a time-dependent ROC analysis. The IPS demonstrated good discriminative ability for all-cause mortality, with AUCs of 0.699 (95% CI: 0.670–0.728), 0.695 (95% CI: 0.672–0.718), and 0.683 (95% CI: 0.663–0.703) at 3, 5, and 7 years, respectively.

Multivariate Cox regression demonstrated a clear trend of increasing mortality risk across IPS quartiles (Table 5). In the unadjusted model, HR increased from 1.000 (reference) in Q1 to 2.964 (95% CI: 2.612–3.364, *P* < 0.001) in Q4. This association remained significant after adjusting for confounders in Model 2 (HR = 2.437, 95% CI: 2.174–2.733, *P* < 0.001), Model 3 (HR = 2.110, 95% CI: 1.887–2.360, *P* < 0.001), and Model 4 (HR = 2.044, 95% CI: 1.802–2.318, *P* < 0.001). Trend tests across all models showed *P*-values < 0.001, indicating a strong positive relationship between IPS and mortality risk.

**Table 5:** Trend of mortality risk across IPS quartiles from multivariate Cox regression.

**Model**	**Quartile1**	**Quartile 2**		**Quartile 3**		**Quartile 4**		***p*-value for trend**
	**HR**	**HR (95% CI)**	***p*** **-value**	**HR (95% CI)**	***p*** **-value**	**HR (95% CI)**	***p*** **-value**	
Model 1	Reference	1.313 (1.139, 1.513)	<0.001^*^	1.814 (1.572, 2.092)	<0.001^*^	2.964 (2.612, 3.364)	<0.001^*^	<0.001^*^
Model 2	Reference	1.180 (1.028, 1.354)	0.019^*^	1.676 (1.461, 1.923)	<0.001^*^	2.437 (2.174, 2.733)	<0.001^*^	<0.001^*^
Model 3	Reference	1.102 (0.958, 1.267)	0.176	1.469 (1.283, 1.682)	<0.001^*^	2.110 (1.887, 2.360)	<0.001^*^	<0.001^*^
Model 4	Reference	1.093 (0.950, 1.257)	0.214	1.459 (1.267, 1.679)	<0.001^*^	2.044 (1.802, 2.318)	<0.001^*^	<0.001^*^

^*^Indicate statistical significance.

HR, hazard ratio; CI, confidence interval; IPS, inflammation prognostic score.

Model 1: adjusted for none; Model 2: adjusted for age, race, PIR, and education; Model 3: adjusted for age, race, PIR, education, BMI, physical activity level, hypertension, diabetes, heart failure, coronary heart disease, cancer, stroke, stroking status, and alcohol consumption; Model 4: adjusted for age, race, PIR, education, BMI, physical activity level, hypertension, diabetes, heart failure, coronary heart disease, cancer, stroke, stroking status, alcohol consumption, urinary albumin, total cholesterol, triglycerides, creatinine, AST, ALT, ALB, RBC, HGB.

### 3.4 Sarcopenia and inflammatory markers

Linear regression analysis revealed relationships between sarcopenia, 12 inflammatory markers, and IPS, as shown in Table 6. Multiple linear regression showed that NEU, MNO, NLR, dNLR, LMR, SIRI, and IPS were consistently and significantly associated with sarcopenia across all models (*P* < 0.05). RDW, WBC, and CRP showed significant associations in the initial models, but these associations weakened or became non-significant in fully adjusted models. LDH and LYM showed no significant associations in any model, while ALP showed a significant association only in Model 4 (*P* = 0.041).

**Table 6:** Multivariate linear regression of sarcopenia, 12 inflammatory markers, and IPS: relationships and associations.

**Variable**	**Model 1**		**Model 2**		**Model 3**		**Model 4**	
	β **(95% CI)**	***p*** **-value**	β **(95% CI)**	***p*** **-value**	β **(95% CI)**	***p*** **-value**	β **(95% CI)**	***p*** **-value**
RDW (%)	0.029 (0.013, 0.045)	<0.001^*^	0.024 (0.007, 0.041)	0.005^*^	0.011 (−0.005, 0.028)	0.189	0.016 (−0.002, 0.034)	0.079
WBC (× 10^9^/L)	0.008 (0.001, 0.015)	0.023^*^	0.008 (0.001, 0.014)	0.035^*^	0.005 (−0.001, 0.011)	0.134	0.005 (−0.001, 0.011)	0.103
NEU (× 10^9^/L)	0.030 (0.015, 0.044)	<0.001^*^	0.028 (0.013, 0.042)	<0.001^*^	0.020 (0.005, 0.035)	0.009^*^	0.021 (0.006, 0.036)	0.005^*^
LYM (× 10^9^/L)	−0.002 (−0.009, 0.005)	0.510	−0.002 (−0.010, 0.004)	0.456	−0.002 (−0.009, 0.004)	0.439	−0.002 (−0.008, 0.004)	0.498
MNO (× 10^9^/L)	0.135 (0.040, 0.230)	0.005^*^	0.115 (0.025, 0.205)	0.013^*^	0.102 (0.020, 0.183)	0.014^*^	0.097 (0.016, 0.179)	0.018^*^
LDH (U/L)	0.000 (−0.000, 0.001)	0.567	0.000 (−0.001, 0.001)	0.487	0.000 (−0.001, 0.000)	0.487	0.000 (−0.001, 0.000)	0.309
ALP (U/L)	0.000 (0.000, 0.001)	0.559	0.000 (0.000, 0.000)	0.941	0.000 (−0.001, 0.000)	0.218	0.000 (0.000, 0.000)	0.041^*^
CRP (mg/dl)	0.027 (0.010, 0.047)	0.008^*^	0.026 (0.006, 0.046)	0.010^*^	0.016 (−0.002, 0.033)	0.077	0.015 (−0.002, 0.033)	0.075
NLR	0.024 (0.011, 0.038)	<0.001^*^	0.023 (0.009, 0.036)	0.001^*^	0.023 (0.010, 0.036)	0.001^*^	0.023 (0.009, 0.037)	<0.001^*^
dNLR	0.042 (0.018, 0.066)	<0.001^*^	0.039 (0.015, 0.064)	0.001^*^	0.038 (0.013, 0.063)	0.003^*^	0.039 (0.013, 0.064)	0.003^*^
LMR	−0.016 (−0.026, −0.006)	0.003^*^	−0.015 (−0.025, −0.004)	0.005^*^	−0.016 (−0.026, −0.005)	0.003^*^	−0.015 (−0.025, −0.005)	0.004^*^
SIRI	0.040 (0.020, 0.060)	<0.001^*^	0.037 (0.017, 0.056)	<0.001^*^	0.036 (0.018, 0.054)	<0.001^*^	0.036 (0.017, 0.054)	<0.001^*^
IPS	0.116 (0.070, 0.162)	<0.001^*^	0.097 (0.050, 0.144)	<0.001^*^	0.062 (0.017, 0.107)	0.006^*^	0.065 (0.019, 0.111)	0.005^*^

^*^Indicate statistical significance.

RDW, red cell distribution width; WBC, white blood cell count; NEU, neutrophil count; LYM, lymphocyte count; MNO, monocyte count; LDH, lactate dehydrogenase; ALP, alkaline phosphatase; CRP, C-reactive protein; NLR, neutrophil-to-lymphocyte ratio; d-NLR, derived neutrophil-to-lymphocyte ratio; LMR, lymphocyte-to-monocyte ratio; SIRI, systemic inflammation response index; IPS, inflammation prognostic score; CI, confidence interval.

Model 1: adjusted for none; Model 2: adjusted for age, race, PIR, and education; Model 3: adjusted for age, race, PIR, education, BMI, physical activity level, hypertension, diabetes, heart failure, coronary heart disease, cancer, stroke, stroking status, and alcohol consumption; Model 4: adjusted for age, race, PIR, education, BMI, physical activity level, hypertension, diabetes, heart failure, coronary heart disease, cancer, stroke, stroking status, alcohol consumption, urinary albumin, total cholesterol, triglycerides, creatinine, AST, ALT, ALB, RBC, HGB.

### 3.5 Mediating effect of inflammatory factors on mortality in sarcopenia patients

**Figure 3:**
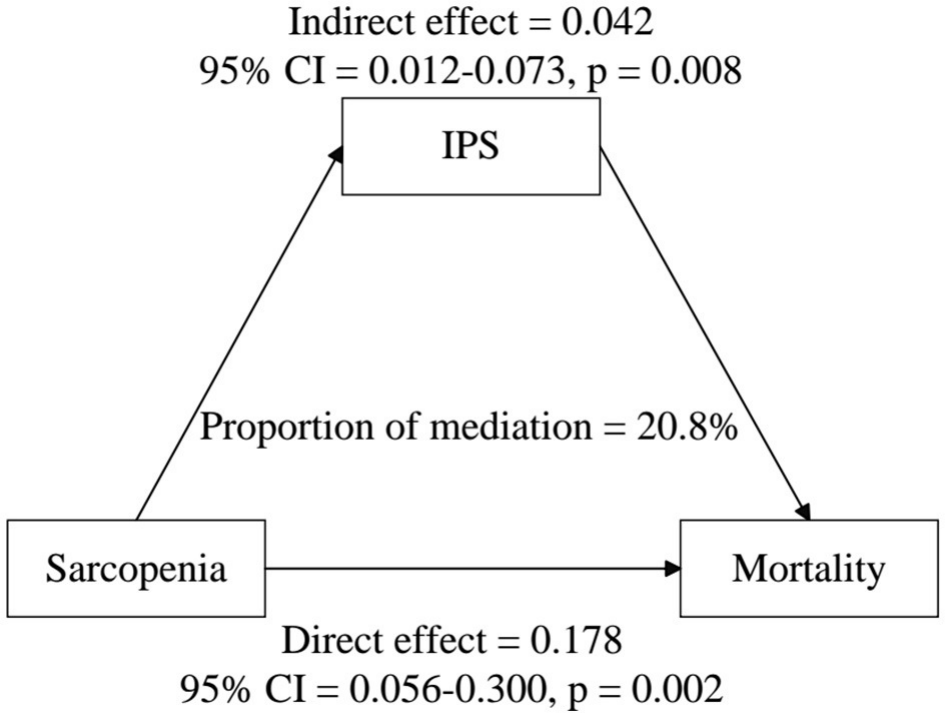
Estimated proportion of the association between sarcopenia and all-cause mortality mediated by IPS. Models were adjusted for age, race, PIR, education, BMI, physical activity level, hypertension, diabetes, heart failure, coronary heart disease, cancer, stroke, stroking status, alcohol consumption, urinary albumin, total cholesterol, triglycerides, creatinine, AST, ALT, ALB, RBC, and HGB.

As shown in Table 7, IPS plays a significant mediating role in the relationship between sarcopenia and mortality. In the unadjusted model (Model 1), the mediation proportion of IPS was 25.7% (95% CI: 0.104–0.410, *P* < 0.001), with a significant Average Causal Mediation Effect (ACME) of 0.093 (95% CI: 0.025–0.161, *P* < 0.001). After adjusting for confounders (Model 2), the mediation proportion decreased to 20.8% (95% CI: 0.001–0.424, *P* < 0.001), while the ACME remained significant at 0.042 (95% CI: 0.012–0.073, *P* = 0.008). These results indicate that IPS mediates ~20.8% of the total effect of sarcopenia on mortality, and its mediating effect persists even after accounting for multiple confounding factors (Figure 3).

**Table 7:** Mediating effect of IPS in the relationship between sarcopenia and mortality: unadjusted and adjusted models.

**Model**	**ACME**			**ADE**			**Total effect**			**Proportion mediated**		
	**Estimate**	**95% CI**	***p*** **-value**	**Estimate**	**95% CI**	***p*** **-value**	**Estimate**	**95% CI**	***p*** **-value**	**Estimate**	**95% CI**	***p*** **-value**
Model 1	0.093	(0.025, 0.161)	<0.001^*^	0.273	(0.106, 0.441)	<0.001^*^	0.366	(0.152, 0.580)	<0.001^*^	0.257	(0.104, 0.410)	<0.001^*^
Model 2	0.042	(0.012, 0.073)	0.008^*^	0.178	(0.056, 0.300)	0.002^*^	0.220	(0.096, 0.344)	<0.001^*^	0.208	(0.001, 0.424)	<0.001^*^

^*^Indicate statistical significance.

ACME, average causal mediation effect; ADE, average direct effect.

Model 1: adjusted for none; Model 2: adjusted for age, race, PIR, education, BMI, physical activity level, hypertension, diabetes, heart failure, coronary heart disease, cancer, stroke, stroking status, alcohol consumption, urinary albumin, total cholesterol, triglycerides, creatinine, AST, ALT, ALB, RBC, HGB.

## 4 Discussion

According to our research, sarcopenia, inflammatory markers, and mortality were closely interconnected. Inflammation, as measured by the IPS, significantly mediated the relationship between sarcopenia and mortality in elderly patients, explaining about 20.8% of this link. IPS was also a strong predictor of mortality, and its predictive power remained robust even after adjusting for confounders. This underscored the crucial role of systemic inflammation in the progression of sarcopenia and its effect on survival outcomes in the elderly population.

Our research aligned with previous studies, confirming that sarcopenia in the elderly was an independent risk factor for all-cause mortality. After adjusting for demographics, chronic diseases, and liver and kidney function, the hazard ratio remained significant at 1.235. Previous studies conducted in diverse populations, including those in intensive care units (28), emergency surgeries (29–31), post-fracture surgeries (32–34), rehabilitation hospitals (35), hemodialysis patients (36), hospitalized elderly (37–39), and care institutions (40, 41), have also demonstrated an association between sarcopenia and increased mortality risk, although the definition of sarcopenia varied across studies. The elevated mortality risk in patients with sarcopenia can be attributed to several factors. Muscle weakness in sarcopenia increases the risk of falls, leading to fractures, hospitalization, and complications such as infections, ultimately contributing to higher mortality (42, 43). Furthermore, elderly patients with sarcopenia undergoing surgery are at heightened risk of postoperative complications, including infections and cardiovascular events (44), which further elevate the likelihood of adverse outcomes.

In this study, we selected 12 commonly used inflammatory markers and identified optimal cut-off points for predicting mortality. Among these markers, SIRI, RDW, NLR, and dNLR demonstrated relatively high predictive capabilities. After adjusting for various confounders, all 12 markers remained significantly associated with all-cause mortality. These findings align with previous studies, which have highlighted the predictive value of inflammatory markers in various populations, including ICU patients (22, 45), post-operative patients with type A aortic dissection (46), and hip fracture patients (19, 20, 47–49), all of whom face elevated mortality risks. Extensive research has demonstrated that markers such as NLR and SIRI are particularly effective in predicting ICU-related mortality, while NLR, MLR, and RDW are key indicators of post-operative mortality. Additionally, studies in community-dwelling elderly populations have confirmed a strong association between NLR and mortality risk, supporting the utility of inflammatory markers in predicting adverse outcomes in older adults. However, Cavdar et al. (50) found that the systemic immune inflammation index (SII), while a significant predictor of mortality in COVID-19 patients under 65, was not significant in older patients. This suggests that chronic low-grade inflammation, commonly observed in elderly populations, may reduce the predictive accuracy of inflammatory markers. Age-related changes in immune function complicate the interpretation of these markers, emphasizing the need for age-specific approaches when applying them in older adults. Therefore, although inflammatory markers are widely used, caution is needed when applying them to certain populations, particularly older adults who may exhibit altered inflammatory responses. Our study addressed this issue by investigating the relationship between multiple inflammatory markers and all-cause mortality in elderly populations, particularly those with sarcopenia. We demonstrated that the IPS score was significantly associated with mortality, highlighting the role of systemic inflammation in sarcopenia-related mortality risk. This finding is crucial, as inflammation not only reflects an acute response but also represents a chronic state that can exacerbate age-related muscle degradation and functional decline, contributing to increased mortality.

Although some studies, such as Chen et al. (20), did not find a significant association between specific markers (e.g., NLR, PLR, SII) and 3-year mortality, these markers are still widely used due to their simplicity and broad applicability in clinical settings. Our approach, however, goes further by integrating multiple markers into the IPS system, providing a more comprehensive assessment of systemic inflammation. LASSO-Cox regression was used to address potential multicollinearity among inflammatory markers, ensuring that only the most predictive variables were retained in the IPS. This integration compensates for potential limitations of individual markers and offers a broader view of inflammation's role in mortality. Our findings confirmed a strong correlation between IPS and mortality, with a significantly increased hazard ratio in the fourth quartile, indicating that higher IPS scores effectively identify high-risk individuals. The IPS system offers a valuable clinical tool for early identification of high-risk groups and for guiding targeted interventions, particularly in older populations where single markers may be insufficient. Although LDH and ALP did not show significant associations with sarcopenia in multivariable models, they were retained in the IPS through a data-driven LASSO-Cox regression procedure based on their predictive contribution to mortality. These markers may reflect underlying tissue damage, metabolic stress, or subclinical organ dysfunction, all of which may be relevant to systemic physiological decline in older adults.

In the linear regression analysis, sarcopenia was significantly associated with several inflammatory markers, particularly NEU, MNO, NLR, dNLR, LMR, and SIRI. Similarly, a cross-sectional study by Nie et al. (51) in elderly populations found that higher SIRI and NLR, along with lower LMR, were associated with sarcopenia. These findings reinforce the notion that inflammation plays a critical role in the pathophysiology of sarcopenia. However, unlike our findings, previous research has linked higher CRP levels with sarcopenia (52–54), reporting elevated CRP levels in individuals with sarcopenia compared to those without while failing to adjust for potential confounding factors. In our study, CRP was initially associated with sarcopenia in unadjusted models, but this relationship weakened after adjusting for confounders. Ozturk et al. (54) also identified NLR as an independent predictor of sarcopenia in the elderly, along with higher WBC levels in participants with sarcopenia. In contrast, the association between WBC and sarcopenia in our study diminished and disappeared after adjustment, suggesting that the relationship between WBC, CRP, and sarcopenia may be mediated by other factors, which were accounted for in our analysis. These inflammatory markers likely reflect the chronic low-grade inflammatory state present in patients with sarcopenia, which is known to impair muscle protein synthesis, increase muscle degradation, and contribute to muscle weakness.

This study is the first to apply formal mediation analysis to investigate the role of systemic inflammation in the relationship between sarcopenia and all-cause mortality. Our findings demonstrate that the inflammatory prognostic score (IPS), derived from routine biomarkers, significantly mediates this association. Notably, the mediating effect remained robust even after adjustment for a wide range of demographic, clinical, and biochemical confounders. The IPS accounted for ~20.8% of the total effect of sarcopenia on mortality, suggesting that inflammation is not merely a coexisting feature, but a key biological pathway contributing to poor outcomes in individuals with sarcopenia. By integrating common inflammatory markers, the IPS provides a comprehensive reflection of the body's inflammatory state. This study confirms that the IPS is independently associated with mortality and mediates the mortality risk in patients with sarcopenia. Given its accessibility, low cost, and predictive utility, the IPS has strong potential for clinical application in risk stratification and early intervention strategies targeting sarcopenia-related adverse outcomes.

This study highlights the mediating role of inflammation in the relationship between sarcopenia and mortality, emphasizing the critical role inflammation plays in the progression of sarcopenia among the elderly. The concept of “inflammaging”—characterized by chronic low-grade inflammation—is a hallmark of aging and is strongly associated with disability, frailty, and premature death (55). Inflammatory mediators such as IL-6 and TNF-α contribute to skeletal muscle protein degradation, impair anabolic signaling, and exacerbate insulin resistance (56, 57). Furthermore, dysfunction in skeletal muscle tissue triggers chronic inflammatory responses, increasing the production of pro-inflammatory cytokines, which further promote catabolism and inhibit muscle protein synthesis, creating a cycle of muscle mass and function decline (58). Chronic inflammation is also linked to diseases such as chronic lung disease, cardiovascular disease, neurological disorders, and cancer (10, 59), all of which heighten mortality risk and worsen the prognosis in the elderly. Together, these findings highlight the importance of targeting inflammation in both the prevention and management of sarcopenia and suggest that inflammation-informed strategies may improve survival and functional outcomes in aging populations.

This study has several limitations. First, sarcopenia was assessed using a single baseline ASM/BMI measurement, which does not capture changes over time, limiting causal interpretation. Second, the diagnosis of sarcopenia was based solely on low muscle mass (ASM/BMI), without incorporating muscle strength or physical performance measures such as grip strength or gait speed. This limitation reflects the data constraints of NHANES 1999–2006 and may lead to underestimation or misclassification of sarcopenia severity and its functional consequences. Future studies should incorporate assessments of muscle strength and physical performance to provide a more complete evaluation of sarcopenia. Third, specific pro-inflammatory cytokines such as IL-6, TNF-α, and IL-1β–known to play direct roles in the pathogenesis of sarcopenia—were not available in the NHANES dataset and, thus could not be analyzed. Instead, we used a range of systemic inflammatory markers that are routinely available in NHANES and constructed a composite IPS to reflect overall inflammatory burden. While this approach provides a useful surrogate, it may not fully capture the cytokine-specific mechanisms underlying sarcopenia. Although we adjusted for many factors, residual confounding may remain due to unmeasured variables such as medication use, nutrition, and immune conditions. In addition, other mechanisms linking sarcopenia and mortality—such as metabolic dysfunction—were not assessed. Future studies with more detailed data are needed to clarify these relationships. Finally, although this study focused on mortality risk, future studies should include other health outcomes, such as quality of life, functional decline, and hospitalization rates, using longitudinal designs to better assess the long-term impact of sarcopenia and inflammation on the elderly population.

## 5 Conclusion

This study, based on a large elderly cohort, confirms a significant association between sarcopenia and increased mortality risk, with inflammation identified as a key mediating factor. Future research should aim to elucidate the specific biological mechanisms through which inflammation contributes to mortality in patients with sarcopenia, and to develop targeted interventions that can reduce this risk. Additionally, the IPS scoring system demonstrates considerable potential for clinical application, offering a practical tool for early identification of high-risk individuals and guiding personalized treatment strategies. Longitudinal studies will be essential to further validate the IPS and assess its long-term utility in improving patient outcomes.

## Data Availability

Publicly available datasets were analyzed in this study. This data can be found at: https://wwwn.cdc.gov/nchs/nhanes/.
